# Stigma and anxiety/depression among Chinese older people living with HIV/AIDS: a moderated mediation model of coping strategies and quality of life

**DOI:** 10.3389/fpubh.2026.1762595

**Published:** 2026-05-14

**Authors:** Lijun Meng, Luxi Duan, Dan Chen, Cui Zhou, Meng Yao, Xingli Li

**Affiliations:** 1 Hunan Cancer Hospital, Changsha, Hunan, China; 2 Hunan Children's Hospital, Changsha, Hunan, China; 3 Department of Epidemiology and Health Statistics, Xiangya School of Public Health, Central South University, Changsha, Hunan, China

**Keywords:** anxiety, depression, HIV/AIDS, quality of life, stigma

## Abstract

**Background:**

Older people living with HIV/AIDS (PLWHA) are increasingly susceptible to depression and anxiety, with HIV-related stigma being a significant contributing factor. However, the mechanisms underlying this association remain poorly understood in this specific population. This study investigates whether coping strategies mediate, and multidimensional quality of life (QOL) moderates, the relationship between stigma and mental health outcomes (depression and anxiety) among older PLWHA.

**Methods:**

A cross-sectional study was conducted involving 254 PLWHA aged ≥50 years. Data were collected through face-to-face interviews, encompassing socio-demographics, HIV-related factors, stigma, QOL (total and dimensional scores), coping strategies (confrontation, avoidance, resignation), and mental health assessed using the PHQ-9 (for depression) and GAD-7 (for anxiety). Mediation and moderated mediation analyses were performed using the PROCESS macro, with coping strategies as mediators and QOL dimensions as moderators.

**Results:**

The positive rate for depressive symptoms (PHQ-9≥10) was 50.4%, and that for anxiety symptoms (GAD-7≥10) was 29.9%. Higher levels of stigma and greater reliance on resignation coping were found to predict more severe symptoms of depression and anxiety. Resignation coping partially mediated the effect of stigma on both anxiety and depression. Furthermore, QOL, particularly its psychosocial and spiritual dimensions, moderated the direct pathway from stigma to anxiety/depression.

**Conclusion:**

HIV-related stigma exacerbates depression and anxiety among older PLWHA, partly via resignation coping, while higher QOL, particularly in psychosocial and spiritual domains, buffers the adverse effects of stigma. Interventions targeting passive coping styles and improving psychosocial and spiritual wellbeing may help mitigate the mental health burden of stigma in this population.

## 1 Introduction

The aging of the global population is reflected in the growing number of older people living with HIV/AIDS (PLWHA) (1). The widespread use of effective antiretroviral therapy (ART) has led to a substantial improvement in life expectancy among PLWHA, which combined with the increasing number of new HIV infections in older adults who engaged in high-risk sexual behaviors, has contributed to the growth of older PLWHA (2, 3). Most previous studies generally adopted the age of 50 years as the cutoff for defining older PLWHA (4, 5). Epidemiological evidence has shown that adults aged 50 years and older represent over 50% of PLWHA in high-income countries, including the United States and Europe (6). A retrospective cohort study from China has revealed that the proportion of older male HIV/AIDS cases increased by 49.4% from 2002 to 2017, whereas the corresponding proportion among females increased by 54.6% (7). Older PLWHA are more vulnerable and face greater challenges due to the combined effects of HIV infection, age-related comorbidities, and geriatric conditions (8, 9). Achieving sustainable long-term health benefits for this growing population remains a current priority in global health research.

Anxiety and depression are the most prevalent psychiatric comorbidities in this particular group (10, 11). Previous studies have demonstrated that the rates of depression and anxiety among PLWHA are both significantly higher than in the general population, with the prevalence of depression ranging from 6 to 41% and anxiety ranging from 3.85 to 45.7% (12, 13). With the combination of a lack of family or social support, HIV infection, and aging, older PLWHA suffer more psychological problems than the general older population (14). Poor psychological wellbeing contributes to the risk of adverse behaviors such as concealment of a positive diagnosis, medication non-adherence, substance abuse, high-risk sexual behaviors, and even suicide, which substantially undermine the achievement of UNAIDS 95-95-95 goals (15). Therefore, it is crucial to recognize the key contributors to anxiety and depression and investigate the potential mechanisms underlying the impact of these factors on anxiety and depression.

HIV-related stigma, defined as the social devaluation, prejudice, and discrimination faced by PLWHA, has been recognized as a significant barrier to achieving sustainable health improvements (16). It exerts multifaceted adverse effects on both physical and psychological wellbeing (17). The persistence of HIV-related stigma is rooted in a complex interplay of factors, including historical associations with marginalized behaviors, fear of transmission fueled by limited health literacy, moral judgments surrounding sexuality or substance use, and sociocultural norms that reinforce exclusion. Despite substantial efforts to mitigate stigma, PLWHA remains highly stigmatized. For instance, our previous study revealed a high prevalence of perceived stigma among older PLWHA in China (18). HIV-related stigma has been strongly associated with numerous negative health outcomes, including depression, anxiety, emotional distress, poor treatment adherence, and insufficient social support (16, 19).

Although stigma is a recognized risk factor for anxiety and depression, not all affected individuals develop these conditions because of various protective and mediating factors. Although the mechanisms linking stigma to mental health outcomes have been studied (20, 21), research specifically examining these relationships among older PLWHA remains limited.

Coping strategies, which refer to the skills that patients use to manage diseases or stressful situations, are commonly categorized into three main types: confrontation, avoidance, and resignation strategies (22). Among these, confrontation, as a positive and adaptive coping style, is associated with better health outcomes and improved wellbeing. In contrast, avoidance and resignation strategies, which are passive coping mechanisms, are linked to the onset of adverse psychological and physical events, ultimately influencing disease outcomes (23). Stigma often functions as a chronic psychosocial stressor that shapes the selection and effectiveness of coping strategies. Specifically, perceived and internalized stigma can severely limit an individual's coping repertoire, promoting reliance on passive strategies such as avoidance and resignation and reducing their ability to adopt adaptive, confrontational approaches (24). The role of coping strategies as a mediating factor is particularly significant because coping mechanisms directly influence psychological adjustment by determining how individuals respond to social stressors such as stigma. These findings emphasize that stigma is positively associated with the use of maladaptive coping styles, and the limited engagement in adaptive coping exacerbates mental health risks. Consequently, stigma may indirectly contribute to anxiety and depression by fostering patterns of passive or disengaged coping, ultimately hindering effective psychological adjustment and wellbeing.

Quality of life (QOL), a multidimensional construct encompassing physical, psychological, and social domains, has emerged as a critical indicator of overall health adaptation. Utilizing QOL as a moderator is vital for identifying the specific life domains that most effectively buffer the psychological impact of stigma, thereby informing the design of targeted interventions. While total QOL scores provide a broad overview, dimensional analysis distinguishes whether physical functioning, psychological wellbeing, social relationships, or spiritual fulfillment serves as the primary protective factor. This detailed analysis not only deepens the theoretical understanding of resilience mechanisms but also facilitates the development of precise support strategies. Previous studies have shown that health-related QOL is negatively correlated with HIV-related stigma, depression, and anxiety (25, 26). Moreover, better QOL is recognized as a key determinant of psychological wellbeing and is instrumental in promoting resilience among PLWHA. Accordingly, this study proposes a moderated mediation model to investigate whether different dimensions of QOL moderate the direct relationship between stigma and psychological distress via coping strategies.

**Figure 1:**
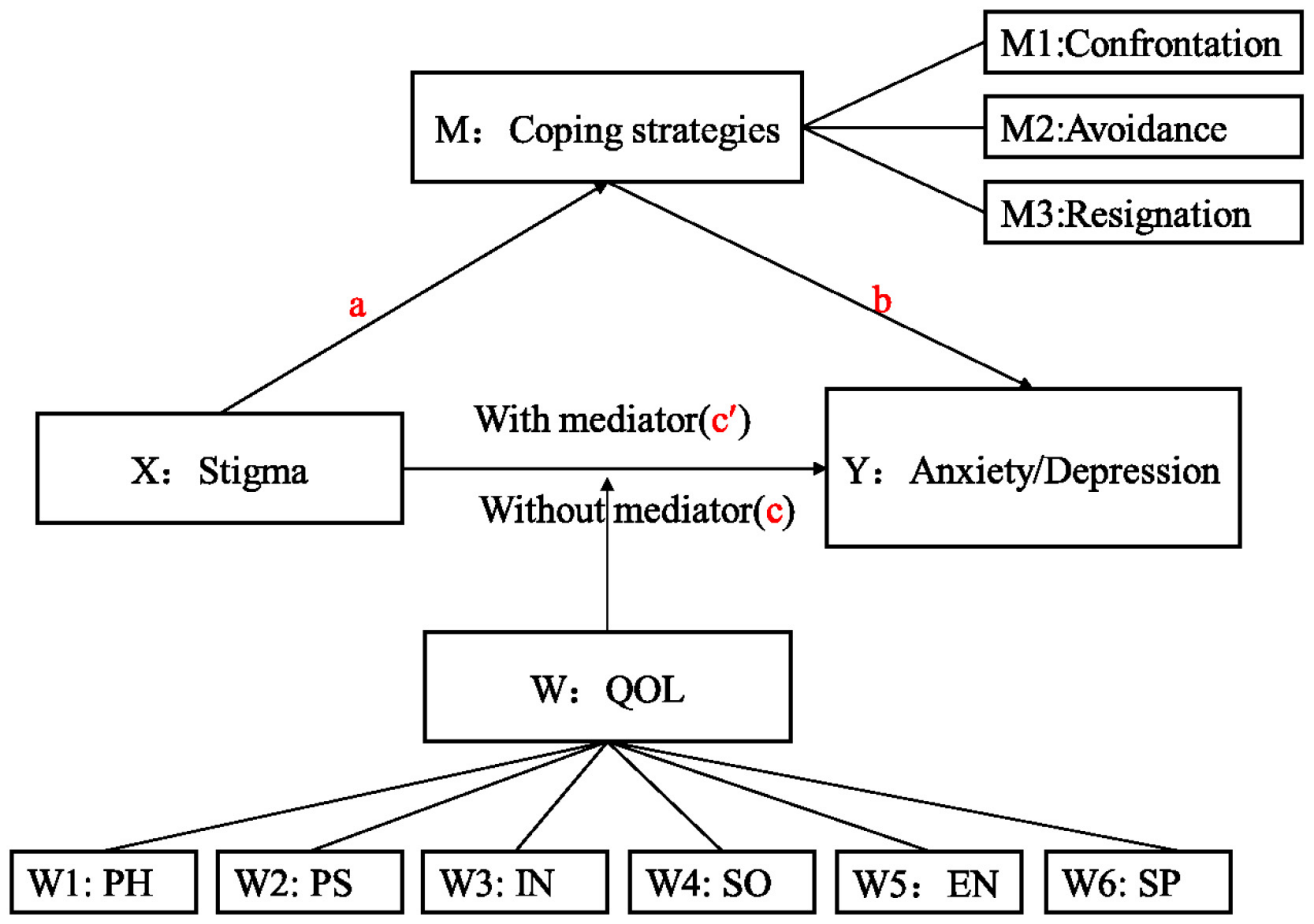
The hypothesis model. QOL, Quality of life; PH, Physical domain; PS, Psychological domain; IN, Independent domain; SO, Social domain; EN, Environmental domain; SP, Spiritual domain.

Taken together, stigma, coping strategies, and QOL play important roles in the recognition of anxiety and depression symptoms. However, the possible impact of these mechanisms on anxiety and depression symptoms in older PLWHA is poorly understood. Therefore, the following hypotheses were proposed: (i) HIV-related stigma may positively and directly contribute to anxiety and depression, (ii) coping strategies may play a mediating role in the association between stigma and depression/anxiety, and (iii) the direct effects of stigma on anxiety and depression are moderated by different dimensions of QOL (Figure 1).

## 2 Materials and methods

### 2.1 Study design and sample

A cross-sectional study was conducted from May to July 2017 in Zhangjiajie, Changde, and Chenzhou cities of Hunan Province, which are in three remote regions of southern China, where a growing incidence of older PLWHA has been observed in recent years. All the participants were recruited by the Hunan HIV/AIDS registry system. The formula used to calculate the sample size was: *n* = $\frac{Z_{\alpha /2}^{2}\times P\times \left(1-P\right)DEFF}{d^{2}},$ where *Z*_α/2_ is the significance test statistic, *P* is the estimated prevalence, *d* is the error margin, and *DEFF* is the design effect. Given the previous empirical research literature and the fact that the sampling was non-random, *P* was set at 50%, α = 0.05, *d* = 0.08, and *DEFF*=1.2 to obtain a sample size of at least 180 respondents. The sample size was appropriately increased, considering the validity of the questionnaire. The criteria for recruitment in this study were as follows. Inclusion criteria: (i) age of at least 50 years and identified with a HIV-positive condition, (ii) no psychiatric medical history of oneself or family, and (iii) mental competence and willingness to cooperate throughout the survey. Exclusion criteria: (i) age < 50 years, (ii) presence of any personal or family psychiatric medical history, (iii) pregnancy or lactation, and (iv) inability to complete the face-to-face interview due to dialect or other communication issues lack of mental competence or unwillingness to cooperate. Due to dialect issues, the entire survey was conducted by trained local researchers through face-to-face interviews. To minimize potential biases associated with face-to-face interviews, several additional measures were implemented. First, all interviewers received standardized training on interview techniques, including maintaining neutral wording, avoiding leading questions, and following a uniform script for each questionnaire item. Second, interviews were conducted in a private, quiet room to ensure confidentiality and reduce social desirability bias. Third, participants were assured that their responses would be anonymized and used solely for research purposes, and they were encouraged to answer as honestly as possible. Fourth, regular quality control checks were performed by the research team, including random audits of completed questionnaires and periodic re-training of interviewers.

### 2.2 Measures

#### 2.2.1 Socio-demographic and HIV-related characteristics

A self-reported questionnaire was employed to collect socio-demographic information and HIV-related factors regarding older PLWHA, such as gender, age, occupation, education level, marital status, living status, annual income, co-morbid chronic diseases, time to diagnosis of being HIV-positive, stage of infection, route of infection, status on antiretroviral therapy, and presence of side effects of therapy.

#### 2.2.2 Stigma

The modified Berger's HIV Stigma Scale (BHSS) was designed to detect the perceived extent of HIV-related stigma, covering four dimensions: personalized stigma, disclosed concerns, negative self-image, and public attitude concerns. With its validity and stability across cultures and ethnic groups, it is broadly recognized as the benchmark measurement tool for assessing HIV stigma among PLWHA (27). The total score was calculated by summing the response values of all items with adjustments for reverse entries. Increasing scores were associated with greater perceived HIV-related stigma. The total stigma score was adopted for the primary analyses due to the substantial correlations among the four subscales and the study's emphasis on the overall effect of stigma. Employing a single total score avoided multicollinearity and facilitated clearer interpretation of the mediation and moderation models.

#### 2.2.3 Depression and anxiety

The Patient Health Questionnaire-9 (PHQ-9) and Generalized Anxiety Disorder-7 (GAD-7), were used to measure depression and anxiety status in the past 2 weeks. The total anxiety and depression scores ranged from 0 to 27 and 0 to 21, respectively, with higher scores reflecting more severe levels of depression/anxiety. Considering the sensitivity and specificity of the scale in different characteristics of the population, a cut-off score of ≥10 was employed to define positive screening status for depressive and anxiety symptoms in the present study (28), which does not equate to a clinical diagnosis according to DSM/ICD criteria.

#### 2.2.4 Coping strategies and QOL

The revised Chinese version of the Medical Coping Modes Questionnaire (MCMQ) was developed to assess the coping strategies of older PLWHA with regard to HIV/AIDS (23), which consisted of 20 items including three subscales: confrontation (eight items), avoidance (seven items), and resignation (five items). Confrontation is a positive coping mode, avoidance is a way of ignoring and minimizing the problem, and resignation is a negative coping mode that increases the psychological burden.

QOL was estimated using a brief version of the WHOQOL-HIV (WHOQOL-HIV BREF) questionnaire, a scale intended to assess the QOL of patients with HIV/AIDS. The scale was comprised six dimensions: QOL in the physical, psychological, independence, social relationships, environment, and spiritual/personal beliefs. Each item was scored on a 5-point Likert scale (1 to 5), of which seven entries required reverse scoring, with higher scores associated with better QOL.

### 2.3 Data analysis

EpiData version 3.1 software was employed to establish the database, and IBM SPSS software (version 25.0) was used for data analysis. Group comparisons were performed using chi-square analysis and *Pearson* correlation analysis was used to assess relationships between variables. Analysis of the mediating (Model 4 of the PROCESS macro) or moderated mediating effect (Model 5 of the PROCESS macro) of the third variable (coping style and QOL) between the explanatory variable (stigma) and outcome variable (depression or anxiety) was performed using the PROCESS macro version 3.4. The effect was estimated using point estimates and 95% bias-corrected bootstrap confidence intervals, where non-zero intervals indicated significance. Moderated mediation was determined using a statistically significant interaction term and simple slope analyses. All analyses were conducted following standard procedures to ensure valid interpretation of the mediating and moderating effects. In addition, covariates (age, gender, annual income, chronic disease status, and route of infection) were controlled for in all models and decentered for the study variables.

## 3 Results

### 3.1 Socio-demographic and HIV-related factor characteristics of participants

A total of 254 participants were included in this study, with a mean age of 60.0 ± 8.0 years (range: 50–84 years). Most participants were male (63.0%), employed or retired (61.4%), married (65.4%), living with others (70.1%), and had an annual income of less than 6,000 RMB (70.9%). In terms of disease characteristics, the total average length of infection since diagnosis was 3.3 years, and 61.8% of patients had progressed to the AIDS stage, with sexual transmission being the predominant route of infection. A vast majority of participants (95.3%) were receiving antiretroviral therapy. The positive rates for depressive symptoms and anxiety symptoms varied among older PLWHA, with an overall positive rate of 50.4% (128/254) for depression and 29.9% (76/254) for anxiety observed in this population. Univariate analysis revealed significant variations in both anxiety and depression among patients of different ages, genders, chronic disease statuses, and routes of infection (Table 1).

**Table 1:** Sample characteristics of older PLWHA and distribution of depression and anxiety.

Characteristic	*N* (%)	Anxiety (*n*, %)		*χ^2^*	Depression (*n*, %)		*χ^2^*
		Yes (*n* = 76)	No (*n* = 178)		Yes (*n* = 128)	No (*n* = 126)	
Age (years old)							
50~	126 (49.6)	45 (35.7)	81 (64.3)	4.002^*^	72 (57.1)	54 (42.9)	4.556^*^
15.6-7.4,-1.3498pt ≥60	128 (50.4)	31 (24.2)	97 (75.8)		56 (43.8)	72 (56.3)	
Gender							
Male	160 (63.0)	39 (24.4)	121 (75.6)	6.342^*^	71 (44.4)	89 (55.6)	6.265^*^
15.6-7.4,-1.3498pt Female	94 (37.0)	37 (39.4)	57 (60.6)		57 (60.6)	37 (39.4)	
Occupation							
Unemployed	98 (38.6)	35 (35.7)	63 (64.3)	2.554	63 (64.3)	35 (35.7)	12.318^**^
15.6-7.4,-1.3498pt Employed/retired	156 (61.4)	41 (26.3)	115 (73.7)		65 (41.7)	91 (58.3)	
Education							
Primary School or less	122 (48.0)	38 (31.1)	84 (68.9)	0.168	67 (54.9)	55 (45.1)	1.922
15.6-7.4,-1.3498pt Junior school or above	132 (52.0)	38 (28.8)	94 (71.2)		61 (46.2)	53 (53.8)	
Marital status							
Single/divorced/others	88 (34.6)	28 (31.8)	60 (68.2)	0.231	49 (55.7)	39 (44.3)	1.506
15.6-7.4,-1.3498pt Married	166 (65.4)	48 (28.9)	118 (71.1)		79 (47.6)	87 (52.4)	
Living status							
Alone	76 (29.9)	18 (23.7)	58 (76.3)	2.012	39 (51.3)	37 (48.7)	0.037
15.6-7.4,-1.3498pt Live with others	178 (70.1)	68 (32.6)	120 (67.4)		89 (50.0)	89 (50.0)	
Annual income (RMB)							
≤ 6,000	180 (70.9)	60 (33.3)	120 (66.7)	3.430	106 (58.9)	74 (41.1)	17.836^**^
15.6-7.4,-1.3498pt >6,000	74 (29.1)	16 (21.6)	58 (78.4)		22 (29.7)	52 (70.3)	
Chronic disease status							
Yes	84 (33.1)	38 (45.2)	46 (54.8)	14.042^**^	55 (65.5)	29 (34.5)	11.421^**^
15.6-7.4,-1.3498pt No	170 (66.9)	38 (22.4)	132 (77.6)		73 (42.9)	97 (57.1)	
Infective stage							
HIV	97 (38.2)	28 (28.9)	69 (71.1)	0.083	47 (48.5)	50 (51.5)	0.236
15.6-7.4,-1.3498pt AIDS	157 (61.8)	48 (30.6)	109 (69.4)		81 (51.6)	76 (48.4)	
Length of infection (year)							
< 1	46 (18.1)	13 (28.3)	33 (71.7)	2.314	22 (47.8)	24 (52.2)	4.117
1~5	150 (59.1)	41 (27.3)	109 (72.7)		70 (46.7)	80 (53.3)	
15.6-7.4,-1.3498pt ≥5	58 (22.8)	22 (37.9)	36 (62.1)		36 (62.1)	22 (37.9)	
Route of infection							
Sexual transmission	247 (97.2)	70 (28.3)	177 (71.7)	10.686^**^	121 (49.0)	126 (51.0)	7.086^**^
15.6-7.4,-1.3498pt Others/unknown	7 (2.8)	6 (85.7)	1 (14.3)		7 (100)	0 (0.0)	
Antiretroviral therapy							
Yes	242 (95.3)	75 (31.0)	167 (69.0)	2.799	125 (51.7)	117 (48.3)	3.249
No	12 (4.7)	1 (8.3)	11 (91.7)		3 (25.0)	9 (75.0)	

^*^p < 0.05; ^**^p < 0.01; Comparison between groups using chi-squared test.

### 3.2 Bivariate correlation analysis between the key variables

The means, standard deviations, and *Pearson*'s correlation coefficients of the key variables were presented Table 2. Bivariate *Pearson* correlation analysis revealed that higher levels of stigma were significantly associated with greater severity of both anxiety (*r* = 0.253, *p* < 0.01) and depression (*r* = 0.297, *p* < 0.01). Resignation coping was positively correlated with anxiety (*r* = 0.354, *p* < 0.01) and depression(*r* = 0.476, *p* < 0.01). However, confrontation (*r* = 0.141, *p* < 0.05) and avoidance coping (*r* = 0.151, *p* < 0.05) demonstrated only weak correlations with anxiety symptoms and were not significantly associated with depression (*p* > 0.05). In addition, QOL was significantly negatively correlated with stigma(*r* = −0.203, *p* < 0.01), anxiety (*r* = −0.647, *p* < 0.01) and depression (*r* = −0.785, *p* < 0.01).

**Table 2:** Matrix of correlation coefficients for stigma, coping strategies, quality of life, anxiety and depression.

Variable	*M*	SD	1	2	3	4	5	6	7
1. Stigma	10.73	4.03	1.000						
2. Confrontation	15.16	4.03	−0.084	1.000					
3. Avoidance	16.44	2.70	0.092	0.199^**^	1.000				
4. Resignation	11.06	4.00	0.416^**^	−0.177^**^	0.051	1.000			
5. QOL	76.94	11.93	−0.203^**^	−0.005	0.04	−0.427^**^	1.000		
6. Anxiety	7.13	5.47	0.253^**^	0.141^*^	0.151^*^	0.354^**^	−0.647^**^	1.000	
7. Depression	10.20	5.87	0.297^**^	0.041	0.063	0.476^**^	−0.785^**^	0.727^**^	1.000

^*^p < 0.05;^**^p < 0.01; M, mean; SD, standard deviation; QOL, Quality of life.

### 3.3 Mediating role of coping strategies in stigma and anxiety/depression

The potential mediating roles of the different coping strategies in both the links from stigma to anxiety/depression were analyzed using PROCESS macro Model 4 (Table 3). For anxiety, the total effect of stigma on anxiety was 0.344 with a 95% confidence interval (*CI*) of [0.180, 0.507] excluding 0, suggesting that stigma was a significant positive predictor of anxiety. After the three coping strategies were included as mediators, the direct effect remained significant. Only the indirect effect through resignation coping was significant, with the indirect effect value of 0.170 [bootstrap 95% *CI*: (0.091, 0.265], while neither confrontation nor resistance had a significant indirect effect. As the direct effect of stigma on anxiety remained significant, resignation coping functioned as a partial mediator, accounting for approximately 49.4% of the total effect.

**Table 3:** Test of the mediating role of coping strategies between stigma and anxiety or depression.

*Paths*	Bootstrap effects					
	Total effect(c)	*Boot 95% CI*	Direct effect	Boot *95% CI*	Indirect effect	Boot *95% CI*
1. Stigma → confrontation → anxiety	0.344	**[0.180, 0.507]**	0.362	**[0.200, 0.524]**	−0.019	[−0.058, 0.011]
2. Stigma → avoidance → anxiety			0.327	**[0.165, 0.490]**	0.016	[−0.006, 0.047]
3. Stigma → resignation → anxiety			0.173	**[0.011, 0.346]**	0.170	**[0.088, 0.262]**
4. Stigma → confrontation → depression	0.432	**[0.259, 0.604]**	0.440	**[0.267, 0.613]**	−0.008	[−0.034, 0.011]
5. Stigma → avoidance → depression			0.427	**[0.254, 0.601]**	0.005	[−0.014, 0.029]
6. Stigma → resignation → depression			0.173	**[0.013, 0.347]**	0.258	**[0.169, 0.365]**

Bold numbers in the table indicated statistically significant at 0.01, which bootstrap interval did not include 0.

The mediation analysis with depression as the dependent variable showed similar results. Bootstrap analysis with 5,000 iterations showed that stigma exerted not only a direct effect on depression, but also an indirect effect through resignation coping. The indirect effect was estimated at 0.258 [boot 95% *CI*: (0.169, 0.365)], accounting for 59.7% of the total effect, supporting the role of resignation coping as a partial mediator of this relationship. Two valid mediation models were developed for stigma and anxiety or depression. These findings indicate that resignation coping significantly mediates the relationship between stigma and psychological distress.

### 3.4 Moderating role of QOL in the mediation model

**Figure 2:**
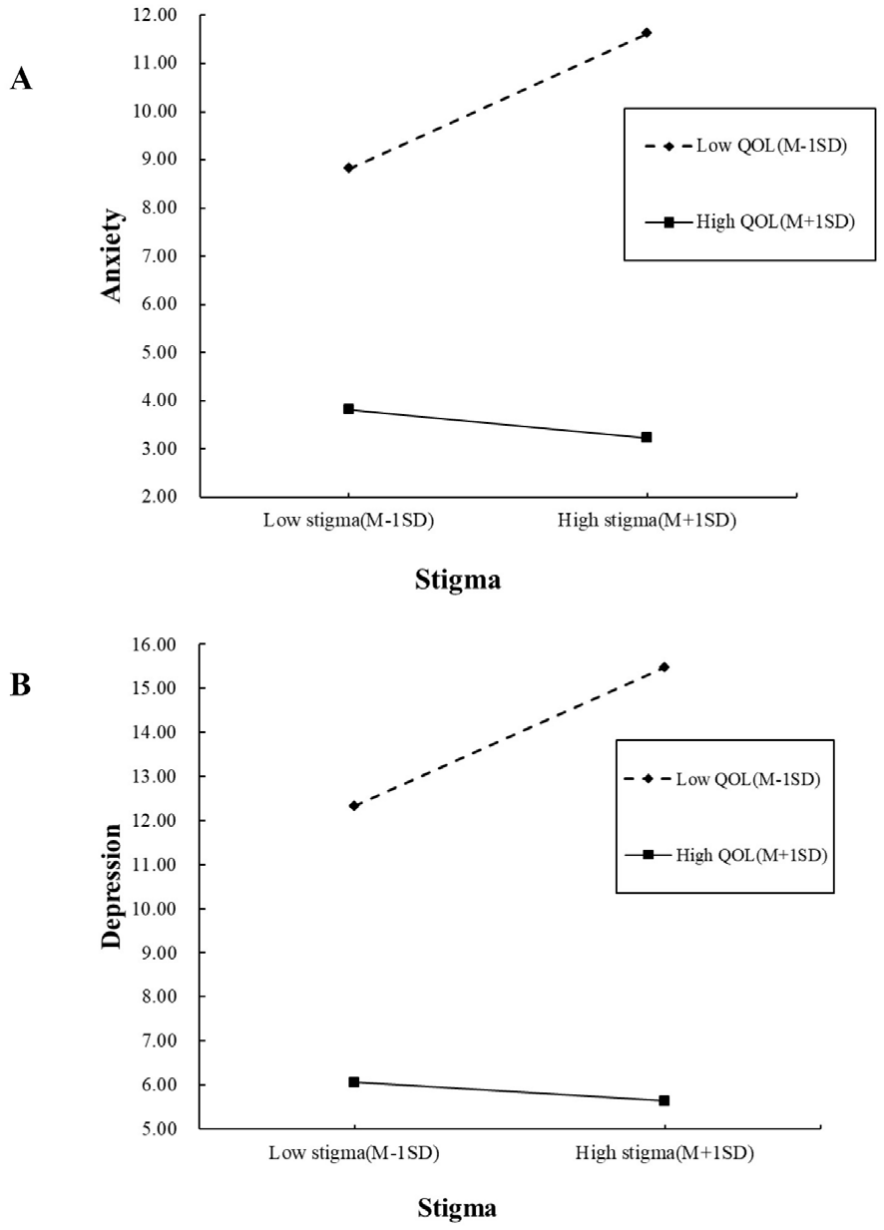
**(A, B)** Simple slope plots of total quality of life as a moderator in the association between stigma and anxiety/depression. QOL, Quality of life.

The results of the moderated mediation analysis were presented in Table 4. To examine the moderating role of the overall and dimensional QOL on the direct effect of stigma on anxiety and depression, a moderated mediation analysis was conducted using Model 5 of the PROCESS macro. The interaction term between stigma and overall QOL significantly predicted both anxiety [β = −0.015, bootstrap 95%*CI*: (−0.027, −0.004)] and depression [β = −0.017, bootstrap 95%*CI*: (−0.026, −0.007)], indicating that QOL moderated the direct effect of stigma on these mental health outcomes. Analysis of QOL in each dimension revealed a significant prediction of anxiety and depression, with moderating effects being significant only in the psychological, social relationship, environmental, and spiritual dimensions. The predictive impact of stigma on anxiety/depression in above- and below-average QOL was further investigated to validate the moderating role. Simple slope analyses revealed that stigma was a significant positive predictor of anxiety/depression at low levels of the total QOL. However, the effect of stigma on anxiety/depression tended to be flat and statistically insignificant at high levels of QOL (Figure 2).

**Table 4:** Results of moderated mediation analysis.

Variable	M: Resignation			Y1: Anxiety			Y2: Depression		
	β	*SE*	*Boot 95% CI*	β	*SE*	*Boot 95% CI*	β	*SE*	*Boot 95% CI*
X: Stigma	0.413	0.057	**[0.301, 0.524]**	0.163	0.894	**[0.025, 0.302]**	0.158	0.059	**[0.042, 0.274]**
M: resignation				0.412	0.088	**[0.239, 0.586]**	0.627	0.089	**[0.451, 0.802]**
W: total QOL				−0.272	0.024	**[−0.319**, **−0.225]**	−0.343	0.020	**[−0.382**, **−0.303]**
W1: PH				−0.745	0.111	**[−0.963**, **−0.526]**	−1.071	0.100	**[−1.269**, **−0.873]**
W2: PS				−1.013	0.101	**[−1.213**, **−0.813]**	−1.433	0.08	**[−1.590**, **−1.270]**
W3: IN				−0.612	0.126	**[−0.860**, **−0.364]**	−1.028	0.116	**[−1.256**, **−0.800]**
W4: SO				−0.437	0.128	**[−0.689**, **−0.185]**	−0.643	0.125	**[−0.889**, **−0.397]**
W5: EN				−0.767	0.125	**[−1.013**, **−0.522]**	−1.050	0.118	**[−1.282**, **−0.818]**
W6: SP				−1.017	0.095	**[−1.204**, **−0.831]**	−0.704	0.105	**–[0.911**, **−0.497]**
X × W				−0.015	0.006	**[−0.027**, **−0.004]**	−0.017	0.005	**[−0.026**, **−0.007]**
X × W1				−0.012	0.028	[−0.068, 0.044]	−0.006	0.026	[−0.057, 0.045]
X × W2				−0.059	0.026	**[−0.110**, **−0.009]**	−0.042	0.02	**[−0.082**, **−0.002]**
X × W3				−0.042	0.031	[−0.103, 0.018]	−0.029	0.028	[−0.085, 0.026]
X × W4				−0.023	0.032	**[−0.085**, **−0.004]**	−0.050	0.031	**[−0.111**, **−0.001]**
X × W5				−0.056	0.028	**[−0.111**, **−0.011]**	−0.042	0.027	**[−0.094**, **−0.021]**
X × W6				−0.026	0.024	**[−0.074**, **−0.020]**	−0.066	0.027	**[−0.120**, **−0.013]**

*SE*, Standard erro*r*; β, Standardized coefficients; QOL, Quality of life; PH, Physical domain; PS, Psychological domain; IN, Independence domain; SO, Social domain; EN, Environment domain; SP, Spirituality domain. Bold numbers in the table indicated statistically significant at 0.05, which bootstrap interval did not include 0.

## 4 Discussion

This study examined the prevalence of anxiety and depression among remote Chinese PLWHA aged ≥50 years and explored the mediating role of coping strategies between stigma and anxiety/depression, as well as how different levels of QOL moderated stigma-induced anxiety and depression through a moderated mediation model. The findings indicated that stigma, resignation coping, and QOL were all significantly associated with anxiety/depression symptoms; however, the effects of these factors differed. Resignation coping mediated the association between stigma and anxiety/depression. Furthermore, the direct effect of the mediation model was moderated by different levels of QOL, with higher QOL buffering the direct effect of stigma on anxiety/depression in older PLWHA. A mediation model of stigma and depression/anxiety incorporating coping strategies and QOL, was successfully constructed to provide theoretical support for alleviating stigma-induced mental health problems.

The results of this contribution implied that depression (50.4%) and anxiety (29.9%) among older PLWHA in China were not optimistic, and the prevalence was greater than those among younger PLWHA (13, 29) and the general older adults (30). Several potential factors may account for the heightened threat of mental health problems in older PLWHA. Research has shown that aging and HIV infection may decrease the human immune response independently or interactively, which may pose substantial psychosocial challenges for older PLWHA (10, 31). In addition, perceived stigma, social discrimination, and loneliness can lead to a deterioration in mental health (32, 33). In general, there was a higher prevalence of anxiety/depression in older PLWHA, which warrants greater attention. Clinically, routine mental health screening should be integrated into HIV care for older PLWHA, and interventions targeting stigma reduction may yield substantial benefits in this population.

Consistent with the findings of previous studies (17), stigma can exacerbate symptoms of depression or anxiety among older PLWHA. A possible explanation for these findings may be interpreted in terms of the mechanisms underlying the psychological problems provoked by both internal and external stigma. PLWHA suffer from tremendous psychological stress due to persistent inequalities in access to health care, employment, and socialization, coupled with restrictions on the availability of treatment modalities and economic pressures (34). In addition, internal stigma leading to the concealment of their condition, fear of losing friends/family and social status may worsen their psychological burden (19). Among older PLWHA, ageism is also a significant contributor to depression, anxiety, and the common co-occurrence of both (35). Therefore, intervention strategies for anxiety and depression in PLWHA should involve the elimination of external and internal stigma, such as providing effective and intensive psychological counseling and adequate social support for patients. Moreover, community-based anti-stigma campaigns targeting both healthcare providers and the general public could help reduce discrimination at the societal level.

We further explored the mediators between stigma and depression or anxiety. Among the three coping strategies, only resignation coping mediated the relationship between stigma and depression/anxiety, which is supported by the theoretical framework. Older PLWHA employed less positive coping strategies and more avoidance and resignation coping strategies. Research evidence supports the idea that negative coping strategies are a hazardous factor for the risk of depression and anxiety disorders (36, 37). To a great extent, older PLWHA who perceive stigma may be prone to surrender to the pressures of HIV infection, and resignation coping would be a countervailing acceptance of various negative emotions that may be associated with poor mental health outcomes more likely (38, 39). Therefore, it is imperative that healthcare facilities evaluate the coping strategies of older PLWHA while providing antiretroviral therapy to improve the physical and mental health outcomes of patients by minimizing the use of resignation coping strategies. Specifically, cognitive-behavioral therapy (CBT) and resilience-training programs that replace resignation coping with active problem-solving and positive reappraisal could be integrated into routine HIV care. Although the present study focused on anxiety and depression, coping strategies may also affect overall mental health, including psychological wellbeing, social functioning, and life satisfaction. Given that resignation coping mediated the relationship between stigma and anxiety/depression, similar mechanisms may extend to broader mental health outcomes. Future research should directly assess overall mental health using comprehensive measures such as the World Health Organization Five Wellbeing Index (WHO-5) or the Mental Health Continuum-Short Form (MHC-SF).

Moderated mediation analysis further indicated that the overall QOL moderated the strength of the relationship between stigma and anxiety/depression mediated by resignation coping. This finding aligns with the stress-buffering model, suggesting that a high overall appraisal of one's life can provide the resilience necessary to mitigate the impact of social adversity (40, 41). The psychological, social, environmental, and spiritual dimensions were significant moderators, whereas the physical and independence dimensions did not reveal a crucial distinction. Protective factors appear to be primarily psychosocial and existential in nature, rather than functional. Specifically, intrinsic resources such as positive self-esteem (psychological), strong social support (social relationships), a safe and accessible environment, and a sense of meaning and purpose (spiritual) are directly effective in counteracting social exclusion and internalized shame associated with stigma. By contrast, being free of pain or able to perform daily tasks independently does not protect individuals against profound psychological damage caused by stigma. This finding has important practical implications, indicating that interventions for older PLWHA should prioritize strengthening psychosocial and existential resources (e.g., peer support groups, meaning-centered therapy) rather than focusing solely on physical function. Mental health interventions for stigmatized groups should not be limited to eliminating stigma or psychological problems, but should adopt a holistic model of connected, supportive and positive interventions to promote mental health wellbeing. Future research could examine the long-term effectiveness of such holistic interventions and explore whether similar moderated mediation patterns exist in other stigmatized populations or across different cultural contexts.

## 5 Limitations

Several limitations of the present study were noted. First, it was limited by the cross-sectional design of our study, which made it difficult to clarify causal relationships. Second, a convenience sampling method was adopted to incorporate participants because of the specificity of the study population, which made our assessment potentially biased owing to the omission of patients with limited mobility or unwillingness to be surveyed. Third, given the presence of multicollinearity and the study's focus on overall effects, the analysis was limited to the total stigma score, with no analysis conducted at the dimensional level. Future research should examine the specific effects of each subscale on mental health. Finally, this study only included older PLWHA; cross-age comparisons with young individuals are needed to identify age-related factors affecting anxiety and depression.

## 6 Conclusion

The findings of this study demonstrate that resignation coping strategies significantly predict HIV-related stigma and anxiety/depression among Chinese older PLWHA, while partially mediating this psychosocial pathway. Furthermore, the adverse impact of stigma on anxiety and depression is alleviated under conditions of high QOL, particularly when the protective effects stem from psychosocial and spiritual domains rather than physical or functional capacities. Interventions grounded in behavioral psychology that actively target and reduce resignation behaviors while simultaneously bolstering multidimensional QOL are essential to disrupt this cycle and mitigate the mental health burden in this vulnerable population.

## Data Availability

The original contributions presented in the study are included in the article/supplementary material, further inquiries can be directed to the corresponding author.
